# Supplementary material for: Social disconnectedness, economic outcomes, and the role of pre-existing mental health conditions: A population-based cohort study
Source: PLOS Ment Health. 2025 May 28;2(5):e0000218. doi: 10.1371/journal.pmen.0000218 (PMC12798343; doi:10.1371/journal.pmen.0000218)
Supplement: S1 Text — (PDF) [file pmen.0000218.s001.pdf]

S1 Text. Supplementary methods

Multiple imputation

We found that partly missing survey data was associated with the investigated monetary outcomes (Methods Table 1). Therefore, at complete case analysis would entail risk of bias (1,2) which can be reduced with multiple imputation given that the included auxiliary variables can predict the missing values. As shown in Methods Table 2, individuals with missing survey data were slightly older and more likely to be out of employment and live alone. We conducted multiple imputation by chained equations (MICE) with predictive mean matching (PMM) using a recommended donor pool of 10 observations (3). As shown in Methods Table 3, survey data on specific scores/responses on loneliness, social isolation, social support, and self-reported mental health conditions were imputed using a range of auxiliary variables including, as recommended (4,5), the outcome and population weights. A total of 15 imputations were conducted with a burn-in of 10. As shown in Methods Table 4, the prevalence of loneliness, social isolation, low social support of complete, and self-reported mental health conditions varied between the complete and imputed data.

Methods Table 1: Quantitative bias analysis among the 158,970 individuals in four regions of Denmark, 2014 & 2018

	N (%)	Excess health care costs (95% CI)	Wage income difference (95% CI)	Excess transfer payments (95% CI)
Included in CCA	139,295 (87.6)	Reference	Reference	Reference
Missing survey data	19,675 (12.4)	€441 (€251 to €630)	€-3,947 (€-4,405 to €-3,488)	€1,983 (€1,812 to €2,154)

CCA: Complete case analysis; CI: Confidence interval. Absolute numbers and proportions are unweighted. The estimates are adjusted for sex, age (included as a natural cubic spline with five knots), year of survey participation, and country of birth and are weighted based on register data to represent the population of the included regions in 2013 and 2017.

Methods Table 2: Baseline characteristics of individuals with missing data in four regions of Denmark, 2013 & 2017

	Included in CCA (N = 139,295)	Missing survey data (N = 19,675)
Age, mean (SD)	47.2 (18.3)	54.0 (21.5)
Women, N (%)	74,976 (50.4)	11,026 (52.9)
Survey participation in 2013 as opposed to 2017, N (%)	27,057 (20.9)	5,487 (26.2)
Born abroad, N (%)	9,901 (11.3)	1,777 (14.8)
Hospital-diagnosed mental health condition, N (%)	8,722 (7.6)	1,393 (8.6)
Psychopharmacological redemption, N (%)	35,951 (25.0)	6,629 (32.7)
Consultation with a private practicing psychiatrist, N (%)	7,482 (5.8)	1,020 (5.7)
Annual health care costs in euro, mean (SD)	2,168.8 (7,530.1)	3,121.4 (10,098.0)
Annual primary wage income in euro, mean (SD)	30,863.7 (35,839.8)	18,559.2 (28,466.4)
Annual transfer payments in euro, mean (SD)	7,881.3 (9,361.9)	12,250.6 (10,561.0)
Living with a partner (self-reported), N (%)	92,244 (60.6)	7,285 (51.7)
Emotional limitations in daily activities (item 6–7 in SF-12), mean (SD)	8.6 (1.9)	8.0 (2.3)
The sum of the Perceived Stress Scale, mean (SD)	12.1 (7.3)	13.7 (7.3)
In education c.f. register data, N (%)	11,329 (11.4)	1,296 (8.7)
In employment c.f. register data, N (%)	80,095 (56.8)	7,269 (37.6)

CCA: Complete case analysis; SF-12: 12-Item Short Form Survey. Absolute numbers are unweighted, whereas means, SDs, and proportions are weighted based on register data to represent the population of the included regions in 2013 and 2017.

Methods Table 3: Variables applied in imputation of survey data among individuals in four regions of Denmark, 2013 & 2017

	N (%) missing	Range	Mean (SD)
**Imputed variables**			
The Three-Item Loneliness Scale, item 1	8,164 (5.1)	1 to 3	1.3 (0.5)
The Three-Item Loneliness Scale, item 2	8,177 (5.1)	1 to 3	1.3 (0.6)
The Three-Item Loneliness Scale, item 3	8,568 (5.4)	1 to 3	1.3 (0.5)
The social isolation index, living alone	8,057 (5.1)	0 to 1	0.2 (0.4)
The social isolation index, out of employment and not enrolled in education	8,901 (5.6)	0 to 1	0.4 (0.5)
The social isolation index, less than monthly contact with friends	8,056 (5.1)	0 to 1	0.1 (0.3)
The social isolation index, less than monthly contact with family	7,152 (4.5)	0 to 1	0.1 (0.3)
The social support item	6,921 (4.4)	1 to 4	1.6 (0.8)
Self-reported mental health condition	11,155 (7.0)	0 to 1	0.1 (0.4)
**Analysis model variables**			
Population weights*	0 (0)	1 to 220	29.0 (19.3)
Age**	0 (0)	16 to 102	51.9 (18.2)
Female	0 (0)	0 to 1	0.5 (0.5)
Born abroad	0 (0)	0 to 1	0.1 (0.3)
Year of survey	0 (0)	2,013 to 2,017	2,016.2 (1.6)
Hospital-diagnosed mental health condition	0 (0)	0 to 1	0.1 (0.2)
Annual health care costs in euro*	0 (0)	-4 to 411,649	2,455.0 (7,921.0)
Annual wage income in euro*	0 (0)	0 to 1,424,090	29,717.0 (36,585.8)
Annual transfer payments in euro*	0 (0)	-3,118 to 64,544	8498.8 (9310.9)
**Auxiliary variables at survey participation**			
Psychopharmacological redemption	0 (0)	0 to 1	0.3 (0.4)
Consultation with private practicing psychiatrist	0 (0)	0 to 1	0.1 (0.2)
Enrolled in education c.f. register data	≤5 (NA)	0 to 1	0.1 (0.3)
In employment c.f. register data	≤5 (NA)	0 to 1	0.5 (0.5)
Cohabitation c.f. register data	≤5 (NA)	0 to 1	0.8 (0.4)
Living with a partner, self-reported	10,555 (6.6)	0 to 1	0.7 (0.5)
Long-term disease, self-reported	7,397 (4.7)	0 to 1	0.4 (0.5)
The sum of the Perceived Stress Scale*	10,273 (6.5)	0 to 40	11.7 (7.1)
Score on getting enough sleep to feel rested	6,250 (3.9)	1 to 3	1.5 (0.7)
Spending time unwanted alone*	7,050 (4.4)	1 to 4	3.2 (0.9)
Evaluation of own health (item 1 in SF-12)*	1,038 (0.7)	1 to 5	2.6 (0.9)
Emotional limitations in daily activities (item 6–7 in SF-12)*	4,795 (3.0)	2 to 10	8.6 (1.9)
Mental health and vitality (item 9–11 in SF-12)*	5,788 (3.6)	3 to 15	9.3 (1.5)
Social contact limited due to physical or mental health (item 12 in SF-12)*	3,053 (1.9)	1 to 5	4.5 (0.9)

SF-12: 12-Item Short Form Survey. Absolute numbers, percentages, means, and SDs are unweighted. The range is shown using means of the 5 lowest and highest values.
* Included in the imputation model as a linear term
** Included in the imputation model as a natural cubic spline with three knots

Methods Table 4: Distribution of complete and imputed data among individuals in four regions of Denmark, 2013 & 2017

	Complete data	Imputed data
Lonely, N (%)	8,616 (7.2)	844 (11.1)
Socially isolated, N (%)	3,662 (3.0)	659 (6.1)
Low social support, N (%)	19,742 (14.6)	1,065 (17.1)
Self-reported mental health condition, N (%)	21,915 (16.3)	1,960 (18.8)

Absolute numbers are unweighted, whereas proportions are weighted based on register data to represent the population of the included regions in 2013 and 2017.

Statistical analysis

Adjustment procedures

Age was included as a time-varying covariate in 1-year incremental age groups and modelled as a natural cubic spline with 5 knots. For analyses according to age, we remodelled age using a natural cubic spline with 3 knots fitted for the specific age group.

Sensitivity analysis on the operationalization of pre-existing mental health conditions

As a sensitivity analysis on the operationalization of mental health conditions, we repeated the interaction analysis with a broader definition of mental health conditions additionally including the following indicators:

i) Redeemed prescription for psychopharmacological treatment in 18 years preceding survey participation recorded in the Danish National Prescription Registry including antipsychotics (N05A except N05AN), antidepressants (N06A), lithium (N05AN), anxiolytics (N05B except N05BA01), medication for ADHD (C02AC02, N06BA02, N06BA04, N06BA09, and N06BA12), and medication for alcohol and opioid dependence (N07BB-N07BB04), based on a recent study (6)

ii) Consultation with a private practicing psychiatrist in 18 years preceding survey participation recorded in the Danish National Health Service Register (7)

iii) Any preceding or current mental health condition self-reported in the Danish National Health Survey

References

1. Bartlett JW, Harel O, Carpenter JR. Asymptotically unbiased estimation of exposure odds ratios in complete records logistic regression. Am J Epidemiol. 2015 Oct 15;182(8):730–6.

2. Hughes RA, Heron J, Sterne JAC, Tilling K. Accounting for missing data in statistical analyses: multiple imputation is not always the answer. Int J Epidemiol. 2019 Aug 1;48(4):1294–304.

3. Morris TP, White IR, Royston P. Tuning multiple imputation by predictive mean matching and local residual draws. BMC Med Res Methodol. 2014 Jun 5;14:75.

4. Wulff JN, Ejlskov L. Multiple imputation by chained equations in praxis: guidelines and review. Electron J Bus. 2017;15(1).

5. White IR, Royston P, Wood AM. Multiple imputation using chained equations: issues and guidance for practice. Stat Med. 2011 Feb 20;30(4):377–99.

6. Kessing LV, Ziersen SC, Caspi A, Moffitt TE, Andersen PK. Lifetime incidence of treated mental health disorders and psychotropic drug prescriptions and associated socioeconomic functioning. JAMA Psychiatry [Internet]. 2023 Jul 12; Available from: https://jamanetwork.com/journals/jamapsychiatry/fullarticle/2806888

7. Andersen JS, Olivarius NDF, Krasnik A. The Danish National Health Service Register. Scand J Public Health. 2011 Jul;39(7 Suppl):34–7.
