# Supplementary material for: Social disconnectedness, economic outcomes, and the role of pre-existing mental health conditions: A population-based cohort study
Source: PLOS Ment Health. 2025 May 28;2(5):e0000218. doi: 10.1371/journal.pmen.0000218 (PMC12798343; doi:10.1371/journal.pmen.0000218)
Supplement: S1 Fig — (PDF) [file pmen.0000218.s002.pdf]

S1 Fig for: *Social disconnectedness, economic outcomes, and the role of pre-existing mental health conditions: a population-based cohort study*

S1 Fig. Flowchart depicting the study population in four regions of Denmark, 2013 and 2017

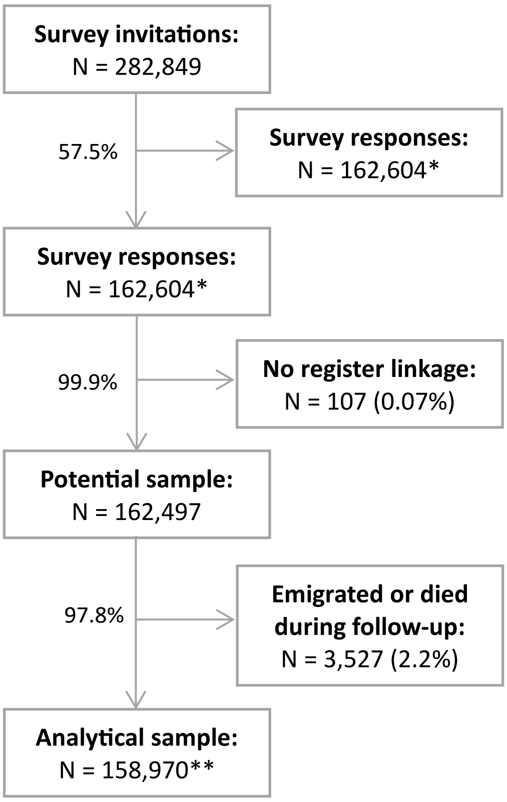

* For a small number (N = 23) of survey participants in 2013, the specific date of survey participation was missing and replaced with the median date.
** For a small number (N ≤ 5) of the analytical sample, data on economic outcomes and/or country of birth was missing and replaced with respectively values of 0 and Denmark.
