# Supplementary material for: Social disconnectedness, economic outcomes, and the role of pre-existing mental health conditions: A population-based cohort study
Source: PLOS Ment Health. 2025 May 28;2(5):e0000218. doi: 10.1371/journal.pmen.0000218 (PMC12798343; doi:10.1371/journal.pmen.0000218)
Supplement: S2 Fig — (PDF) [file pmen.0000218.s003.pdf]

S2 Fig. The first, second, and third quartile of annual healthcare costs, wage income, and transfer payments according to each indicator of social disconnectedness in four regions of Denmark, 2014 & 2018

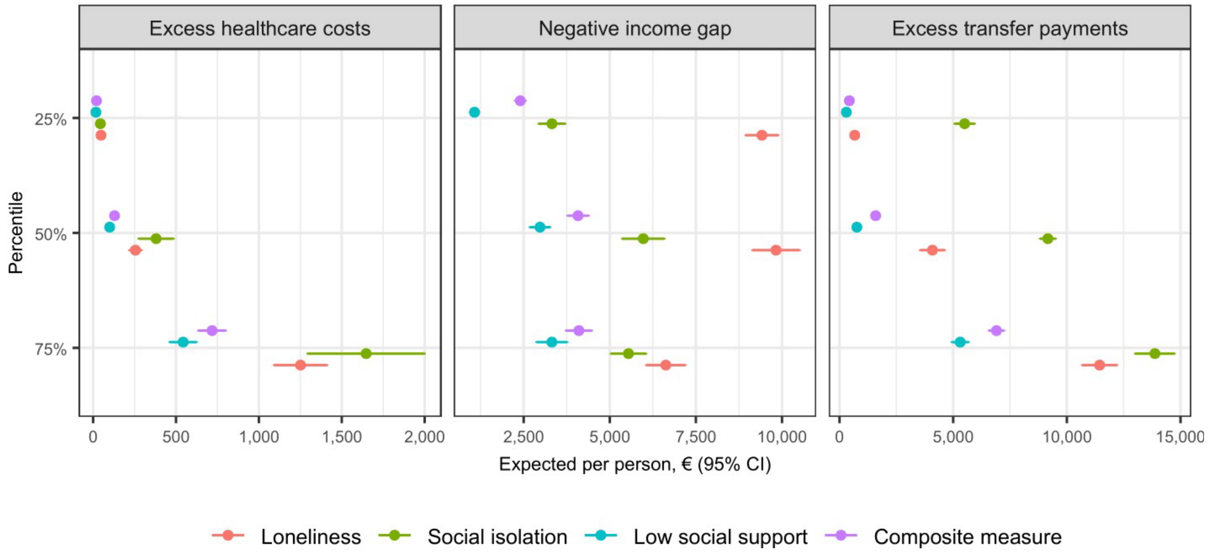

CI: Confidence interval. The colours indicate the different indicators of social disconnectedness. Missing data was imputed using multiple imputation by chained equations, and the results are weighted based on register data to represent the population of the included regions in 2013 and 2017. Please note that the estimation of standard errors and thus the confidence intervals are not completely accurate for these quartile regression models as it was not possible to apply Stata's svy command which takes into account the sampling in the survey design. The estimates represent values in 2018 and are adjusted for sex, age (included as a natural cubic spline with five knots), year of survey participation, and country of birth.
