# Supplementary material for: Social disconnectedness, economic outcomes, and the role of pre-existing mental health conditions: A population-based cohort study
Source: PLOS Ment Health. 2025 May 28;2(5):e0000218. doi: 10.1371/journal.pmen.0000218 (PMC12798343; doi:10.1371/journal.pmen.0000218)
Supplement: S4 Fig — (PDF) [file pmen.0000218.s005.pdf]

S4 Fig for: *Social disconnectedness, economic outcomes, and the role of pre-existing mental health conditions: a population-based cohort study*

S4 Fig. Sex-specific interaction between each indicator of social disconnectedness and pre-existing hospital-diagnosed mental health conditions on annual healthcare costs, wage income, and transfer payments in four regions of Denmark, 2014 & 2018

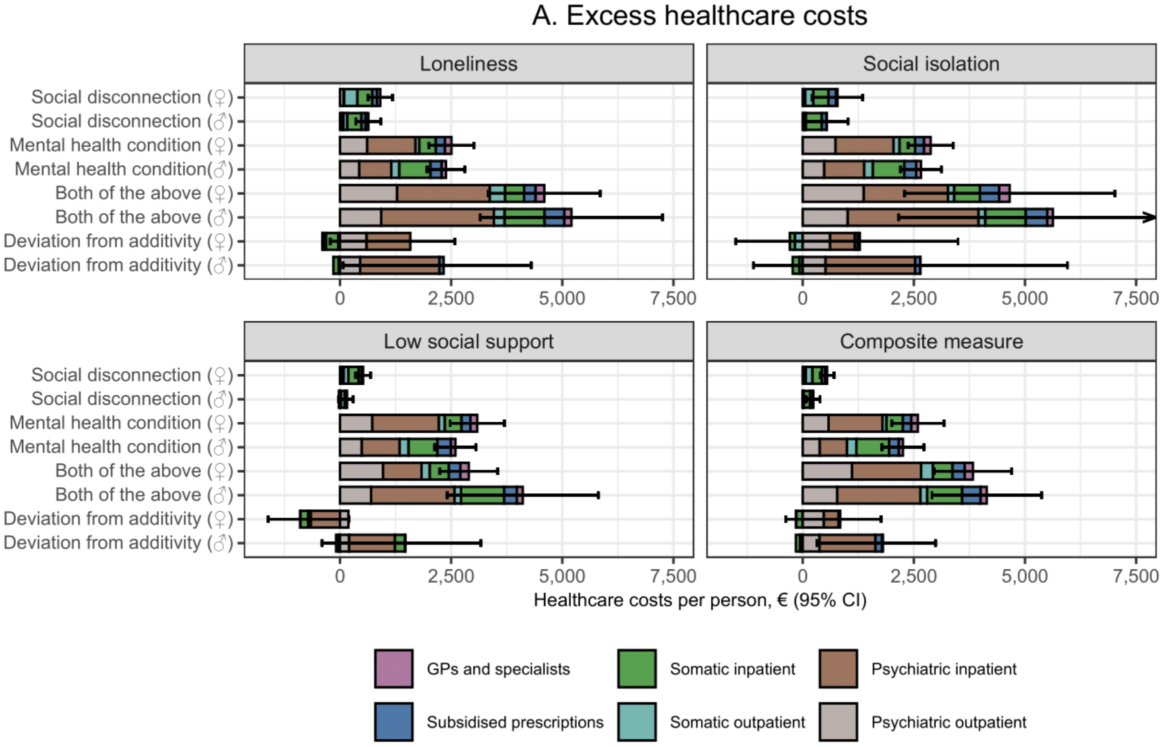

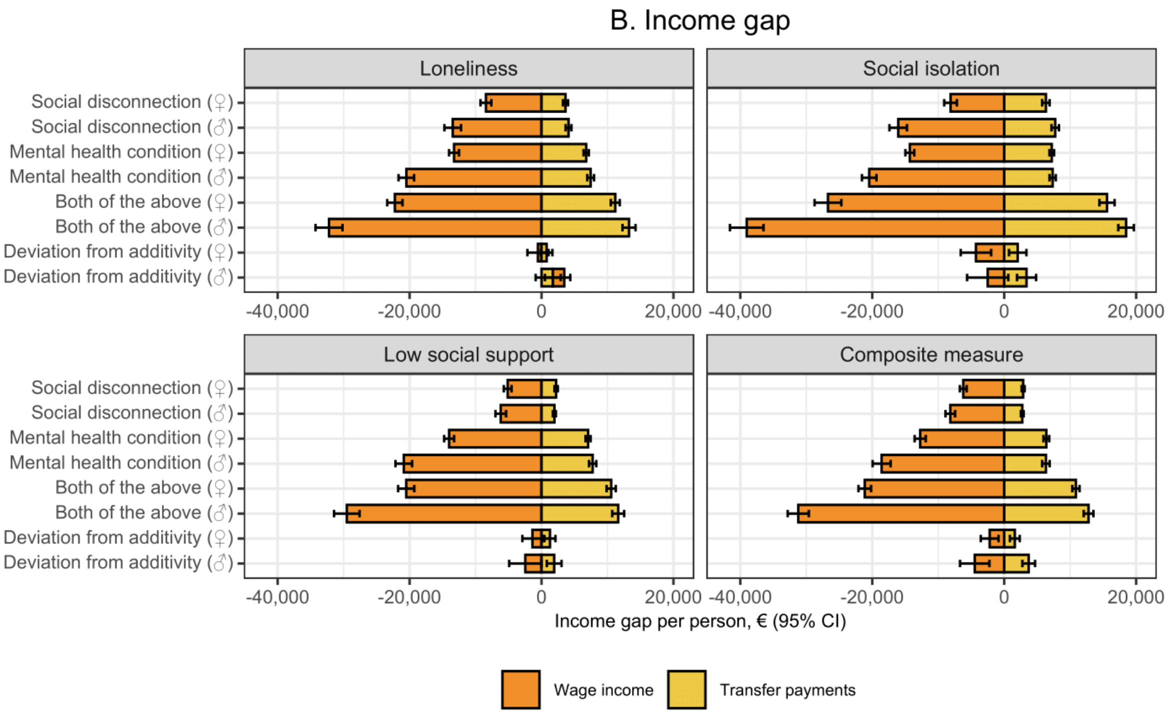

♀: Sex-specific strata of women; ♂: Sex-specific strata of men; CI: Confidence interval; GPs: General practitioners. The colours indicate the contributions of different cost categories. Missing data was imputed using multiple imputation by chained equations, and the results are weighted based on register data to represent the population of the included regions in 2013 and 2017. The estimates represent values in 2018 and are adjusted for age (included as a natural cubic spline with five knots), year of survey participation, and country of birth.
