# Supplementary material for: Social disconnectedness, economic outcomes, and the role of pre-existing mental health conditions: A population-based cohort study
Source: PLOS Ment Health. 2025 May 28;2(5):e0000218. doi: 10.1371/journal.pmen.0000218 (PMC12798343; doi:10.1371/journal.pmen.0000218)
Supplement: S1 Table — (PDF) [file pmen.0000218.s006.pdf]

S1 Table. Overview of prior studies assessing social disconnectedness and economic outcomes

Author, publication year, country	Study design (period)	Study population	Measure(s) of social disconnectedness	Economic outcome(s)	Finding(s)
**Health care costs**					
Landeiro et al. 2016, Portugal (1)	Cohort with follow-up from admission to discharge (2012–2013)	288 patients aged 75 and above admitted with a proximal femoral fracture	**Social isolation** was assessed with the Lubben social network scale of 10 items, which concerns social contacts with family and friends.	Delayed discharges were assessed by reviewing daily the medical records of each patient to check if they had been declared medically fit for discharge by the medical team. Delayed discharges were calculated as the difference between the time a patient was deemed medically fit for discharge and the actual time of discharge.	Adjusting for other covariates, patients who were at a high risk of social isolation before admission to hospital were more likely to have a delay in their discharge than patients with a low risk of social isolation (odds ratio: 3.5). Patients with moderate risk of social isolation spent, on average, an additional 1.5 days of delayed discharge in hospital compared to patients with a low risk of social isolation, holding all else constant, while those with high risk spent, on average, an additional 2.6 days. The additional costs per patient corresponding to these extra days are €532 for the moderate risk group and €905 for the high risk group, per patient.
Meisters et al. 2021, Netherlands (2)	Cohort (2016–2017)	341,376 adults aged 19 years and older from a population-based sample invited to the Health Survey of the Public Health Service 2016.	**Loneliness** based on the 11-item de Jong Gierveld scale. Loneliness is subsequently categorized as follows: "not lonely" (scores between 0 and 2, reference group), "somewhat lonely" (scores between three and 8), "severe loneliness" (scores of nine or 10), and "very severe loneliness" (score of 11).	Based on information from Statistics Netherlands, 1) general practitioners, 2) mental healthcare, 3) pharmaceutical, 4) specialized healthcare, and 5) total healthcare expenditure for the year 2017	Controlling for demographic, socioeconomic, and lifestyle-related factors, loneliness was indirectly (via poorer health) associated with higher expenditure in all categories. In fully adjusted models, it showed a direct association with higher expenditure for general practitioners and mental healthcare (0.5 and 11.1%, respectively). The association with mental healthcare expenditure was stronger in younger than in older adults (for ages 19–40, the contribution of loneliness represented 61.8% of the overall association).
Shaw et al. 2017, USA (3)	Cohort (2006–2012)	5,270 adults continuously enrolled in Original Medicare – Parts A or B and aged ≥65 years from a population-based sample invited in 2006, 2008, or 2010	**Loneliness** was measured using the Three-Item Loneliness Scale. **Social isolation** using a scaled measure of social connectedness, based on normalized values of several survey questions capturing social network and interaction.	Health care cost based on information from Medicare beneficiary summary files	Social isolation predicted greater spending, $1,644 per beneficiary annually, whereas loneliness predicted reduced spending, −$768 per beneficiary annually. Increased spending was concentrated in inpatient and nursing-home care.
**Wage income and transfer payments**					
Bergh et al. 2007, Sweden (4)	Cohort (1999–2003)	132 frequent attenders and 531 normal attenders in primary health care aged below 60 years	**Social support** using an attachment scale as a functional measure of perceived emotional support provided by close friends and family, and instrumental social support (called social integration) as a structural measure of peripheral social ties available for specific functions such as belongingness, tangible support and appraisal support	Based on information from the NHI register on the participants' long-term sick leave and new disability pensions	Emotional support was associated with reduced odds of long-term sick leave and new disability pensions among both frequent and normal attenders in primary health care, whereas instrumental social support was only associated with reduced odds of long-term sick leave and new disability pensions among frequent attenders.

S1 Table for: *Social disconnectedness, economic outcomes, and the role of pre-existing mental health conditions: a population-based cohort study*

**Author, publication year, country**	**Study design (period)**	**Study population**	**Measure(s) of social disconnectedness**	**Economic outcome(s)**	**Finding(s)**
Bergström et al. 2021, Sweden (5)	Cohort with 2 years follow-up (2006–2011)	274 people with rheumatoid arthritis of working age (18–63 years) from the Swedish early RA cohort TIRA-2	**Social support** using a question on whether they received support from family and friends. This information was marked on two scales, one for family and one for friends: "Do you receive practical and/or emotional support from family?" and "Do you receive practical and/or emotional support from friends?"	Data relating to sickness absence were retrieved from the Swedish Social Insurance Agency as the number of days with absence from work during year 1 and year 2 after diagnosis.	Higher perceived support from family and friends at baseline was significantly associated with increased odds of sickness absence during the first, but not the second, year after diagnosis.
Gustafsson et al. 2013, Sweden (6)	Cohort (1991–2011)	53,920 from a population-based sample of women and men interviewed in annual Swedish Surveys of Living Conditions between 1990 and 2007	**Social isolation** based on cohabitation status, frequency of social contacts, and presence of close a friend.	Disability pensions from the Swedish Social Insurance Agency's database	Associations between social isolation and future disability pension were found for grants due to mental health condition diagnoses and younger individuals (20-39 years), also after control for age, year of interview, sociodemographic conditions, and self-reported longstanding illness.
Gustafsson et al., 2014, Sweden (7)	Cohort (1991–2003)	10,936 women born between 1960 and 1979 from a population-based sample interviewed in annual Swedish Surveys of Living Conditions between 1990 and 2002	**Social isolation** measured using the question: "In general how often do you meet with friends, acquaintances or relatives? Do not include current neighbors or workmates".	Disability pensions from the Swedish Social Insurance Agency's database	An increased risk of receiving a disability pension was found among women who had sparse contacts with others.
Kivimäki et al. 1997, Finland (8)	Cohort (1990–1995)	763 local government employees	**Social isolation** measured by 6 items on the number of "important" others from each of the social role domains: spouse, close relative, friend, coworker, some other individual, and nobody (mentioned as social support)	Sickness absence data were gathered from the local government's occupational health care unit register.	Poor social support was not a risk for absence once the effects of baseline absence and potential confounders were taken into account. Without adjustment, however, poor social support was a risk factor.
Lauzier et al. 2008, Cananda (9)	Cohort (2023–2024)	459 breast cancer patients with a paying job during the month before diagnosis	**Social support** was measured using questions on the numbers of individuals available as confidants, who could provide practical assistance and who were close to the respondent and expressed affection for her.	Information on wage losses collected by three telephone interviews	Annual wages loss was associated with a lower level of social support.
Melchior et al. 2003, France (10)	Cohort (1994–2000)	9,631 men and 3,595 women from France's national gas and electricity company (the GAZEL cohort)	**Social isolation** based on four types of social contacts (children, parents/in laws, other family and friends) giving an equal weight to family and non-family ties and divided the distribution into quartiles.<br>**Social support** assessed with an indicator of personal social support as a sum of four items assessing emotional and instrumental help.	Sick absence spells based on information from the social insurance department of the company	Low social support, but not fewer social contacts, were associated with greater incidence of sickness absence spells after adjustment for age. After further adjustments, both measures were a with greater incidence of sickness absence spells.
Morris et al. 2020, 14 countries (11)	Cohort with repeated measures (2013 and 2015)	10,154 adults aged 50-65 years who were working and without work limitations from the Survey of Health, Aging, and Retirement in Europe	**Loneliness** measured using the Three-Item Loneliness Scale	Self-reported work disability based on whether the respondent answers affirmatively to the question: "Do you have any health problem or disability that limits the kind or amount of paid work you can do?" and report not being employed at follow-up.	Loneliness was a significantly predictor of work disability at follow-up (odds ratio: 1.25) in a simple model controlling just for country affiliation. After all adjustments, loneliness still predicts work disability at follow-up (odds ratio: 1.12).

S1 Table for: *Social disconnectedness, economic outcomes, and the role of pre-existing mental health conditions: a population-based cohort study*

**Author, publication year, country**	**Study design (period)**	**Study population**	**Measure(s) of social disconnectedness**	**Economic outcome(s)**	**Finding(s)**
Rael et al. 1995, England (12)	Cohort (1985–1990)	4,202 employees, aged 35-55 years at baseline, from non-industrial departments of the British civil service	**Social support** as three types of received social support over the past 12 months (confiding/emotional, practical, and negative aspects of support) from the person nominated as closest on the "close persons" questionnaire. **Social isolation** was measured with questions about the frequency and number of contacts with relatives, friends, and social groups. Lack of contact in six spheres of relationships (e.g., friends) formed an additive score in a social isolation scale, with a high score reflecting more isolation.	Sickness absence records based on information from the civil service pay centres	Contrary to expectations, high levels of confiding/emotional support from the "closest person" predicted higher levels of both short and long spells of sickness absence, suggesting that high levels of confiding/emotional support may encourage illness behaviour. Social network measures showed a consistent but less striking pattern. Higher levels of practical support from the closest person were associated with higher rates of long spells for women, but not men. Increased levels of negative aspects of social support resulted in higher rates of sickness absence.
Sinokki et al. 2010, Finland (13)	Cohort (2000–2006)	3,414 employees aged 30 to 64 years at baseline from a population-based sample.	**Social support** in private life with four items "On whose help can you really count when you feel exhausted and need relaxation?," "Who do you think really cares about you no matter what happened to you?," "Who can really make you feel better when you feel down?," and "From whom do you get practical help when needed?" reflecting different ways of giving support.	Disability pensions extracted from the registers of the Finnish Centre for Pensions	Low social support in private life was related to 1.94-fold odds of subsequent disability pension (95% CI for odds ratio, 1.35 to 2.78) compared with high support in an unadjusted model. However, after adjustment for sociodemographic factors, neither of these associations remained statistically significant.
Tubach et al. 2002, France (14)	Cohort with repeated measures (1994 and 1996)	2,236 individuals from the GAZEL cohort who were working in the French national electricity and gas company in 1989 and who were still working in 1996 and had answered both the 1994 and 1996 questionnaires.	**Loneliness** assessed with the French version of the Nottingham Health Profile (described as social isolation)	Self-reported sick leave of 8 or more days due to low back pain	In an unadjusted analysis, the risk of sick leave due to low back pain is slightly higher among individuals with a higher loneliness score (risk ratio 1.1). However, in the multivariate adjusted model, the odds are substantially lower among individuals with a higher loneliness score (odds ratio: 0.5).
Van Hoffen et al. 2020, Netherlands (15)	Cohort (2010–2014)	53,833 workers who participated in surveys between 2010 and 2013 and were not on sickness absence at baseline	**Social support** from family and friends was assessed with 3 items (Can you count on the support of partner/family/friends when you have some difficulty at work? Is work at home taken out of your hands if you are busier at work? Do you feel appreciated by your partner/family/friends?)	Long-term sickness absence (≥6 weeks) due to mental health conditions, recorded in an occupational health service register	In the prediction model, social support predicted lower odds for being on long-term sickness absence due to mental health conditions (odds ratio: 0.91).

Based on a literature search in the NCBI PubMed Database on the 16th of January 2024 (1,312 hits) with inclusion of longitudinal or cross-sectional studies reporting on the association between one of loneliness, social isolation, and social support and one of healthcare costs, income, and transfer payments. Studies that reported exclusively on workplace social support or healthcare utilization were excluded.

References

1. Landeiro F, Leal J, Gray AM. The impact of social isolation on delayed hospital discharges of older hip fracture patients and associated costs. Osteoporos Int. 2016 Feb;27(2):737–45.

2. Meisters R, Westra D, Putrik P, Bosma H, Ruwaard D, Jansen M. Does Loneliness Have a Cost? A Population-Wide Study of the Association Between Loneliness and Healthcare Expenditure. Int J Public Health. 2021;66:581286.

3. Shaw JG, Farid M, Noel-Miller C, Joseph N, Houser A, Asch SM, et al. Social Isolation and Medicare Spending: Among Older Adults, Objective Social Isolation Increases Expenditures while Loneliness Does Not. J Aging Health. 2017 Oct;29(7):1119–43.

4. Bergh H, Baigi A, Månsson J, Mattsson B, Marklund B. Predictive factors for long-term sick leave and disability pension among frequent and normal attenders in primary health care over 5 years. Public Health. 2007 Jan 1;121(1):25–33.

5. Bergström M, Dahlström Ö, Thyberg I, Björk M. The role of support from significant others in the association between disease-related factors and sickness absence in early rheumatoid arthritis: a longitudinal study. Scandinavian Journal of Rheumatology. 2021 Nov 2;50(6):427–34.

6. Gustafsson K, Aronsson G, Marklund S, Wikman A, Floderus B. Does Social Isolation and Low Societal Participation Predict Disability Pension? A Population Based Study. PLOS ONE. 2013 Nov 6;8(11):e80655.

7. Gustafsson K, Aronsson G, Marklund S, Wikman A, Hagman M, Floderus B. Social integration, socioeconomic conditions and type of ill health preceding disability pension in young women: a Swedish population-based study. Int J Behav Med. 2014 Feb;21(1):77–87.

8. Kivimäki M, Vahtera J, Pentti J, Thomson L, Griffiths A, Cox T, et al. Psychosocial factors predicting employee sickness absence during economic decline. Journal of Applied Psychology. 1997 Dec;82(6):858–72.

9. Lauzier S, Maunsell E, Drolet M, Coyle D, Hébert-Croteau N, Brisson J, et al. Wage Losses in the Year After Breast Cancer: Extent and Determinants Among Canadian Women. JNCI: Journal of the National Cancer Institute. 2008 Mar 5;100(5):321–32.

10. Melchior M, Niedhammer I, Berkman L, Goldberg M. Do psychosocial work factors and social relations exert independent effects on sickness absence? A six year prospective study of the GAZEL cohort. J Epidemiol Community Health. 2003 Apr;57(4):285–93.

11. Morris ZA. Loneliness as a Predictor of Work Disability Onset Among Nondisabled, Working Older Adults in 14 Countries. J Aging Health. 2020;32(7–8):554–63.

12. Rael EG, Stansfeld SA, Shipley M, Head J, Feeney A, Marmot M. Sickness absence in the Whitehall II study, London: the role of social support and material problems. J Epidemiol Community Health. 1995 Oct;49(5):474–81.

13. Sinokki M, Hinkka K, Ahola K, Gould R, Puukka P, Lönnqvist J, et al. Social support as a predictor of disability pension: the Finnish Health 2000 study. J Occup Environ Med. 2010 Jul;52(7):733–9.

14. Tubach F, Leclerc A, Landre MF, Pietri-Taleb F. Risk Factors for Sick Leave Due to Low Back Pain: A Prospective Study. Journal of Occupational and Environmental Medicine. 2002 May;44(5):451.

15. van Hoffen MFA, Norder G, Twisk JWR, Roelen CAM. Development of Prediction Models for Sickness Absence Due to Mental Disorders in the General Working Population. J Occup Rehabil. 2020 Sep 1;30(3):308–17.
