# Supplementary material for: Social disconnectedness, economic outcomes, and the role of pre-existing mental health conditions: A population-based cohort study
Source: PLOS Ment Health. 2025 May 28;2(5):e0000218. doi: 10.1371/journal.pmen.0000218 (PMC12798343; doi:10.1371/journal.pmen.0000218)
Supplement: S2 Table — (PDF) [file pmen.0000218.s007.pdf]

S2 Table. Total and differences in healthcare costs, wage income, and transfer payments according to each indicator of social disconnectedness in four regions of Denmark, 2014 & 2018

	Subgroup			
	**Loneliness**	**Social isolation**	**Low social support**	**Composite measure**
**Total health care costs (95% CI)**	€3,503 (€3,177 to €3,829)	€4,676 (€4,123 to €5,230)	€2,690 (€2,534 to €2,846)	€2,924 (€2,778 to €3,071)
GPs and specialists (95% CI)	€301 (€292 to €311)	€354 (€340 to €369)	€260 (€254 to €265)	€272 (€267 to €277)
Subsidised prescriptions (95% CI)	€255 (€223 to €287)	€437 (€399 to €475)	€202 (€189 to €215)	€230 (€214 to €245)
Somatic inpatient (95% CI)	€1,015 (€897 to €1,133)	€1,755 (€1,525 to €1,986)	€973 (€894 to €1,052)	€1,008 (€939 to €1,076)
Somatic outpatient (95% CI)	€934 (€844 to €1,024)	€1,297 (€1,155 to €1,439)	€884 (€833 to €935)	€922 (€873 to €970)
Psychiatric inpatient (95% CI)	€631 (€376 to €886)	€568 (€123 to €1,013)	€210 (€107 to €314)	€296 (€195 to €397)
Psychiatric outpatient (95% CI)	€366 (€319 to €413)	€265 (€194 to €336)	€160 (€140 to €181)	€198 (€177 to €218)
**Total income (95% CI)**	€29,871 (€29,387 to €30,355)	€24,502 (€24,073 to €24,931)	€34,608 (€34,207 to €35,010)	€32,757 (€32,426 to €33,089)
Wage income (95% CI)	€17,418 (€16,818 to €18,019)	€3,657 (€3,148 to €4,166)	€24,074 (€23,588 to €24,561)	€21,238 (€20,833 to €21,643)
Transfer payments (95% CI)	€12,453 (€12,165 to €12,740)	€20,845 (€20,492 to €21,198)	€10,534 (€10,350 to €10,719)	€11,519 (€11,355 to €11,683)
**Excess health care costs (95% CI)**	€1,674 (€1,343 to €2,005)	€1,515 (€938 to €2,093)	€561 (€397 to €725)	€828 (€671 to €985)
Excess GPs and specialists (95% CI)	€90 (€81 to €99)	€48 (€34 to €63)	€38 (€33 to €44)	€48 (€43 to €53)
Excess subsidised prescriptions (95% CI)	€146 (€114 to €178)	€195 (€156 to €234)	€63 (€49 to €78)	€97 (€81 to €112)
Excess somatic inpatients (95% CI)	€373 (€251 to €495)	€421 (€185 to €657)	€193 (€109 to €276)	€215 (€140 to €290)
Excess somatic outpatients (95% CI)	€213 (€121 to €304)	€98 (€-48 to €244)	€46 (€-11 to €103)	€83 (€29 to €137)
Excess psychiatric inpatients (95% CI)	€547 (€289 to €805)	€516 (€51 to €980)	€114 (€8 to €221)	€227 (€121 to €334)
Excess psychiatric outpatients (95% CI)	€306 (€258 to €354)	€237 (€163 to €312)	€106 (€84 to €128)	€158 (€136 to €180)
**Income gap (95% CI)**	€-8,013 (€-8,528 to €-7,498)	€-7,318 (€-7,942 to €-6,694)	€-4,361 (€-4,770 to €-3,952)	€-5,379 (€-5,736 to €-5,021)
Wage income difference (95% CI)	€-13,466 (€-14,123 to €-12,809)	€-16,295 (€-17,124 to €-15,467)	€-7,207 (€-7,702 to €-6,711)	€-9,253 (€-9,688 to €-8,818)
Excess transfer payments (95% CI)	€5,453 (€5,171 to €5,736)	€8,977 (€8,583 to €9,372)	€2,845 (€2,666 to €3,025)	€3,875 (€3,713 to €4,037)

CI: Confidence interval; GPs: General practitioners. Missing data was imputed using multiple imputation by chained equations, and the results are weighted based on register data to represent the population of the included regions in 2013 and 2017. The total estimates are calculated using marginal standardization to the distribution of covariates among individuals with each indicator of social disconnectedness (loneliness, social isolation, low social support, and the composite measure, respectively). The estimates represent values in 2018 and are adjusted for sex, age (included as a natural cubic spline with five knots), year of survey participation, and country of birth.
