# Supplementary material for: Social disconnectedness, economic outcomes, and the role of pre-existing mental health conditions: A population-based cohort study
Source: PLOS Ment Health. 2025 May 28;2(5):e0000218. doi: 10.1371/journal.pmen.0000218 (PMC12798343; doi:10.1371/journal.pmen.0000218)
Supplement: S3 Table — (PDF) [file pmen.0000218.s008.pdf]

S3 Table: Sex-specific differences in annual healthcare costs, wage income, and transfer payments according to each indicator of social disconnectedness in four regions of Denmark, 2014 & 2018

	Women				Men			
	**Loneliness**	**Social isolation**	**Low social support**	**Composite measure**	**Loneliness**	**Social isolation**	**Low social support**	**Composite measure**
**Excess health care costs (95% CI)**	€1,744 (€1,336 to €2,152)	€1,401 (€734 to €2,067)	€674 (€490 to €858)	€988 (€774 to €1,202)	€1,572 (€1,034 to €2,109)	€1,590 (€694 to €2,485)	€465 (€206 to €724)	€667 (€439 to €895)
Excess GPs and specialists (95% CI)	€97 (€83 to €110)	€68 (€42 to €94)	€54 (€45 to €63)	€62 (€54 to €70)	€81 (€69 to €93)	€32 (€16 to €49)	€25 (€18 to €32)	€34 (€28 to €40)
Excess subsidised prescriptions (95% CI)	€134 (€111 to €157)	€210 (€161 to €260)	€81 (€63 to €98)	€100 (€85 to €115)	€163 (€97 to €229)	€183 (€125 to €240)	€49 (€27 to €70)	€93 (€66 to €121)
Excess somatic inpatients (95% CI)	€334 (€207 to €461)	€372 (€34 to €710)	€233 (€133 to €333)	€230 (€143 to €318)	€415 (€191 to €639)	€450 (€125 to €775)	€162 (€33 to €290)	€198 (€80 to €315)
Excess somatic outpatients (95% CI)	€300 (€152 to €449)	€163 (€-81 to €407)	€109 (€26 to €192)	€165 (€80 to €250)	€111 (€14 to €207)	€48 (€-130 to €225)	€-7 (€-85 to €72)	€7 (€-63 to €78)
Excess psychiatric inpatients (95% CI)	€511 (€209 to €813)	€336 (€-59 to €731)	€49 (€-41 to €138)	€218 (€75 to €362)	€577 (€145 to €1,009)	€653 (€-109 to €1,416)	€168 (€-16 to €352)	€230 (€77 to €383)
Excess psychiatric outpatients (95% CI)	€368 (€293 to €443)	€251 (€107 to €395)	€149 (€112 to €185)	€212 (€175 to €250)	€226 (€172 to €279)	€224 (€154 to €293)	€69 (€44 to €94)	€104 (€81 to €128)
**Income gap (95% CI)**	€-6,027 (€-6,565 to €-5,489)	€-3,274 (€-3,974 to €-2,574)	€-3,535 (€-3,969 to €-3,100)	€-4,198 (€-4,572 to €-3,825)	€-10,838 (€-11,753 to €-9,924)	€-10,565 (€-11,517 to €-9,612)	€-5,126 (€-5,796 to €-4,456)	€-6,651 (€-7,251 to €-6,052)
Wage income difference (95% CI)	€-11,256 (€-11,966 to €-10,546)	€-11,059 (€-12,014 to €-10,104)	€-6,589 (€-7,150 to €-6,027)	€-8,148 (€-8,634 to €-7,661)	€-16,655 (€-17,801 to €-15,510)	€-20,442 (€-21,698 to €-19,186)	€-7,807 (€-8,599 to €-7,014)	€-10,482 (€-11,190 to €-9,774)
Excess transfer payments (95% CI)	€5,229 (€4,876 to €5,582)	€7,785 (€7,241 to €8,329)	€3,054 (€2,796 to €3,312)	€3,949 (€3,721 to €4,177)	€5,817 (€5,374 to €6,260)	€9,877 (€9,323 to €10,432)	€2,681 (€2,432 to €2,929)	€3,831 (€3,605 to €4,056)

CI: Confidence interval; GPs: General practitioners. Missing data was imputed using multiple imputation by chained equations, and the results are weighted based on register data to represent the population of the included regions in 2013 and 2017. The estimates represent values in 2018 and are adjusted for age (included as a natural cubic spline with five knots), year of survey participation, and country of birth.
