# Supplementary material for: Social disconnectedness, economic outcomes, and the role of pre-existing mental health conditions: A population-based cohort study
Source: PLOS Ment Health. 2025 May 28;2(5):e0000218. doi: 10.1371/journal.pmen.0000218 (PMC12798343; doi:10.1371/journal.pmen.0000218)
Supplement: S4 Table — (PDF) [file pmen.0000218.s009.pdf]

S4 Table. Age-specific differences in annual healthcare costs, wage income, and transfer payments according to each indicator of social disconnectedness in four regions of Denmark, 2014 & 2018

	16–25y (N = 17,907)	26–35y (N = 16,734)	36–45y (N = 23,528)	46–55y (N = 30,230)	56–65y (N = 29,521)	66–75y (N = 27,669)	≥76y (N = 13,381)
Loneliness							
**Excess health care costs (95% CI)**	€1,303 (€618 to €1,987)	€1,566 (€926 to €2,207)	€1,851 (€1,187 to €2,515)	€2,172 (€904 to €3,439)	€1,929 (€1,145 to €2,713)	€1,850 (€953 to €2,747)	€845 (€68 to €1,623)
Excess GPs and specialists (95% CI)	€57 (€40 to €75)	€68 (€48 to €88)	€102 (€79 to €125)	€129 (€102 to €156)	€123 (€99 to €148)	€89 (€58 to €121)	€69 (€29 to €109)
Excess subsidised prescriptions (95% CI)	€110 (€-7 to €228)	€61 (€31 to €91)	€182 (€119 to €246)	€175 (€132 to €219)	€230 (€170 to €290)	€230 (€158 to €301)	€116 (€58 to €174)
Excess somatic inpatients (95% CI)	€99 (€-67 to €266)	€131 (€-61 to €322)	€474 (€47 to €902)	€425 (€192 to €657)	€710 (€301 to €1,119)	€717 (€116 to €1,319)	€642 (€32 to €1,252)
Excess somatic outpatients (95% CI)	€65 (€-9 to €139)	€155 (€-39 to €349)	€359 (€78 to €639)	€218 (€64 to €373)	€299 (€-105 to €702)	€710 (€245 to €1,175)	€-117 (€-508 to €274)
Excess psychiatric inpatients (95% CI)	€556 (€-21 to €1,133)	€741 (€244 to €1,238)	€391 (€120 to €661)	€960 (€-202 to €2,123)	€385 (€47 to €724)	€12 (€-109 to €133)	€102 (€-70 to €273)
Excess psychiatric outpatients (95% CI)	€414 (€303 to €525)	€410 (€303 to €517)	€343 (€174 to €511)	€264 (€160 to €369)	€182 (€92 to €271)	€92 (€42 to €142)	€33 (€-8 to €75)
**Income gap (95% CI)**	€-2,049 (€-2,807 to €-1,290)	€-9,776 (€-11,069 to €-8,482)	€-14,606 (€-16,121 to €-13,091)	€-16,023 (€-17,465 to €-14,580)	€-9,477 (€-10,643 to €-8,311)	€2,731 (€2,076 to €3,386)	€1,975 (€1,411 to €2,539)
Wage income difference (95% CI)	<u>€-3,560 (€-4,501 to €-2,618)</u>	€-14,411 (€-16,064 to €-12,758)	€-22,047 (€-23,956 to €-20,137)	€-25,600 (€-27,438 to €-23,762)	€-17,692 (€-19,218 to €-16,166)	€-1,017 (€-1,512 to €-521)	€-202 (€-294 to €-109)
Excess transfer payments (95% CI)	€1,511 (€1,090 to €1,932)	€4,635 (€3,967 to €5,303)	€7,441 (€6,627 to €8,255)	€9,577 (€8,780 to €10,375)	€8,215 (€7,398 to €9,031)	€3,748 (€3,238 to €4,257)	€2,177 (€1,613 to €2,740)
Social isolation							
**Excess health care costs (95% CI)**	€975 (€-372 to €2,321)	€3,285 (€781 to €5,789)	€2,920 (€1,421 to €4,419)	€3,643 (€230 to €7,056)	€1,920 (€864 to €2,976)	€832 (€164 to €1,500)	€-99 (€-694 to €497)
Excess GPs and specialists (95% CI)	€99 (€26 to €173)	€88 (€11 to €166)	€141 (€88 to €195)	€121 (€69 to €174)	€58 (€28 to €87)	€1 (€-22 to €24)	€-1 (€-30 to €29)
Excess subsidised prescriptions (95% CI)	€171 (€-67 to €409)	€110 (€-16 to €236)	€224 (€90 to €358)	€296 (€182 to €410)	€307 (€215 to €399)	€174 (€81 to €266)	€90 (€43 to €137)
Excess somatic inpatients (95% CI)	€670 (€-459 to €1,798)	€453 (€-223 to €1,129)	€368 (€-122 to €859)	€710 (€96 to €1,324)	€723 (€133 to €1,312)	€454 (€-44 to €951)	€-63 (€-509 to €382)
Excess somatic outpatients (95% CI)	€56 (€-207 to €319)	€261 (€-301 to €824)	€184 (€-147 to €516)	€313 (€-103 to €729)	€109 (€-294 to €513)	€168 (€-141 to €478)	€-138 (€-427 to €150)
Excess psychiatric inpatients (95% CI)	€-124 (€-291 to €43)	€1,488 (€-607 to €3,583)	€1,170 (€109 to €2,231)	€1,815 (€-1,382 to €5,012)	€486 (€3 to €969)	€1 (€-93 to €95)	€-0 (€-59 to €58)
Excess psychiatric outpatients (95% CI)	€103 (€-66 to €272)	€884 (€480 to €1,288)	€832 (€162 to €1,502)	€387 (€146 to €627)	€237 (€101 to €374)	€34 (€6 to €63)	€15 (€-28 to €57)
**Income gap (95% CI)**	€-4,285 (€-6,726 to €-1,845)	€-17,373 (€-21,049 to €-13,698)	€-24,400 (€-27,009 to €-21,791)	€-23,883 (€-25,694 to €-22,071)	€-12,001 (€-13,195 to €-10,808)	€3,219 (€2,809 to €3,629)	€2,725 (€2,311 to €3,140)
Wage income difference (95% CI)	€-7,828 (€-10,756 to €-4,900)	€-27,613 (€-32,260 to €-22,966)	€-41,543 (€-44,821 to €-38,266)	€-41,233 (€-43,523 to €-38,943)	€-24,546 (€-26,001 to €-23,091)	€-2,017 (€-2,263 to €-1,771)	€-206 (€-293 to €-119)
Excess transfer payments (95% CI)	€3,542 (€1,591 to €5,493)	€10,239 (€8,138 to €12,341)	€17,144 (€15,488 to €18,799)	€17,351 (€16,093 to €18,609)	€12,545 (€11,659 to €13,431)	€5,236 (€4,865 to €5,608)	€2,931 (€2,518 to €3,344)
Low social support							
**Excess health care costs (95% CI)**	€351 (€83 to €620)	€662 (€284 to €1,039)	€475 (€160 to €791)	€788 (€234 to €1,341)	€761 (€380 to €1,141)	€536 (€50 to €1,023)	€93 (€-394 to €580)
Excess GPs and specialists (95% CI)	€22 (€9 to €34)	€41 (€26 to €56)	€52 (€37 to €66)	€43 (€30 to €57)	€57 (€43 to €70)	€23 (€7 to €39)	€5 (€-18 to €28)
Excess subsidised prescriptions (95% CI)	€4 (€-24 to €32)	€53 (€15 to €92)	€35 (€10 to €60)	€57 (€33 to €81)	€113 (€80 to €146)	€134 (€58 to €211)	€75 (€36 to €114)

S4 Table for: *Social disconnectedness, economic outcomes, and the role of pre-existing mental health conditions: a population-based cohort study*

	16–25y (N = 17,907)	26–35y (N = 16,734)	36–45y (N = 23,528)	46–55y (N = 30,230)	56–65y (N = 29,521)	66–75y (N = 27,669)	≥76y (N = 13,381)
Excess somatic inpatients (95% CI)	€12 (€-108 to €132)	€-1 (€-92 to €90)	€191 (€-42 to €424)	€189 (€32 to €347)	€375 (€134 to €616)	€445 (€76 to €815)	€243 (€-136 to €623)
Excess somatic outpatients (95% CI)	€73 (€13 to €133)	€204 (€45 to €364)	€26 (€-106 to €158)	€99 (€-11 to €209)	€54 (€-126 to €234)	€-56 (€-259 to €147)	€-234 (€-448 to €-19)
Excess psychiatric inpatients (95% CI)	€65 (€-124 to €254)	€163 (€-107 to €433)	€73 (€-25 to €170)	€285 (€-195 to €764)	€114 (€-21 to €248)	€-29 (€-108 to €51)	€-18 (€-61 to €26)
Excess psychiatric outpatients (95% CI)	€176 (€98 to €253)	€201 (€127 to €275)	€99 (€51 to €146)	€115 (€63 to €166)	€49 (€12 to €85)	€18 (€-1 to €37)	€21 (€-12 to €54)
**Income gap (95% CI)**	€-948 (€-1,614 to €-283)	€-5,972 (€-7,170 to €-4,773)	€-7,174 (€-8,438 to €-5,910)	€-9,374 (€-10,566 to €-8,181)	€-4,084 (€-5,044 to €-3,123)	€1,843 (€1,461 to €2,225)	€1,193 (€841 to €1,546)
Wage income difference (95% CI)	€-1,342 (€-2,142 to €-542)	€-8,948 (€-10,409 to €-7,486)	€-10,301 (€-11,779 to €-8,824)	€-13,972 (€-15,398 to €-12,546)	€-7,981 (€-9,187 to €-6,775)	€-614 (€-958 to €-271)	€-101 (€-233 to €31)
Excess transfer payments (95% CI)	€394 (€86 to €702)	€2,976 (€2,438 to €3,514)	€3,128 (€2,645 to €3,611)	€4,599 (€4,112 to €5,085)	€3,897 (€3,405 to €4,389)	€2,457 (€2,176 to €2,738)	€1,294 (€952 to €1,637)
Composite measure							
**Excess health care costs (95% CI)**	€896 (€498 to €1,295)	€994 (€626 to €1,361)	€922 (€586 to €1,257)	€1,024 (€526 to €1,522)	€942 (€581 to €1,303)	€582 (€169 to €995)	€2 (€-408 to €411)
Excess GPs and specialists (95% CI)	€38 (€27 to €50)	€51 (€37 to €64)	€63 (€50 to €77)	€65 (€52 to €78)	€61 (€49 to €73)	€21 (€7 to €36)	€8 (€-12 to €28)
Excess subsidised prescriptions (95% CI)	€59 (€-4 to €122)	€57 (€27 to €86)	€86 (€56 to €116)	€103 (€79 to €128)	€146 (€115 to €177)	€145 (€85 to €205)	€93 (€60 to €126)
Excess somatic inpatients (95% CI)	€63 (€-41 to €167)	€83 (€-33 to €199)	€239 (€32 to €446)	€272 (€115 to €429)	€339 (€129 to €549)	€362 (€59 to €664)	€159 (€-162 to €480)
Excess somatic outpatients (95% CI)	€77 (€24 to €131)	€183 (€57 to €309)	€127 (€-18 to €272)	€126 (€25 to €227)	€161 (€-49 to €372)	€61 (€-126 to €248)	€-296 (€-477 to €-115)
Excess psychiatric inpatients (95% CI)	€387 (€54 to €719)	€359 (€84 to €633)	€202 (€66 to €338)	€323 (€-103 to €749)	€149 (€22 to €276)	€-31 (€-114 to €51)	€18 (€-34 to €70)
Excess psychiatric outpatients (95% CI)	€272 (€202 to €342)	€262 (€197 to €326)	€204 (€126 to €282)	€135 (€88 to €181)	€86 (€53 to €119)	€24 (€7 to €41)	€19 (€-4 to €43)
**Income gap (95% CI)**	€-1,502 (€-2,082 to €-922)	€-7,601 (€-8,649 to €-6,553)	€-9,901 (€-11,070 to €-8,732)	€-11,640 (€-12,739 to €-10,541)	€-5,901 (€-6,764 to €-5,039)	€2,188 (€1,857 to €2,519)	€1,819 (€1,519 to €2,118)
Wage income difference (95% CI)	€-2,462 (€-3,168 to €-1,755)	€-11,183 (€-12,475 to €-9,891)	€-14,615 (€-16,000 to €-13,229)	€-17,864 (€-19,180 to €-16,548)	€-11,451 (€-12,536 to €-10,366)	€-927 (€-1,220 to €-634)	€-145 (€-249 to €-42)
Excess transfer payments (95% CI)	€960 (€673 to €1,247)	€3,582 (€3,111 to €4,053)	€4,714 (€4,244 to €5,183)	€6,224 (€5,767 to €6,681)	€5,549 (€5,094 to €6,004)	€3,115 (€2,874 to €3,356)	€1,964 (€1,671 to €2,257)

CI: Confidence interval; GPs: General practitioners. Missing data was imputed using multiple imputation by chained equations, and the results are weighted based on register data to represent the population of the included regions in 2013 and 2017. The estimates represent values in 2018 and are adjusted for sex, age (included as a natural cubic spline with three knots), year of survey participation, and country of birth.
