# Supplementary material for: Social disconnectedness, economic outcomes, and the role of pre-existing mental health conditions: A population-based cohort study
Source: PLOS Ment Health. 2025 May 28;2(5):e0000218. doi: 10.1371/journal.pmen.0000218 (PMC12798343; doi:10.1371/journal.pmen.0000218)
Supplement: S5 Table — (PDF) [file pmen.0000218.s010.pdf]

S5 Table. Interaction between each indicator of social disconnectedness and pre-existing hospital-diagnosed mental health conditions on annual healthcare costs, wage income, and transfer payments in four regions of Denmark, 2014 & 2018

	Social disconnectedness	Mental health condition	Both	Deviation from additivity
Loneliness				
**Excess health care costs (95% CI)**	€783 (€590 to €976)	€2,470 (€2,130 to €2,810)	€4,872 (€3,752 to €5,993)	€1,619 (€427 to €2,812)
Excess GPs and specialists (95% CI)	€68 (€58 to €77)	€130 (€120 to €140)	€187 (€165 to €208)	€-11 (€-37 to €14)
Excess subsidised prescriptions (95% CI)	€103 (€83 to €123)	€226 (€200 to €252)	€331 (€222 to €439)	€2 (€-112 to €116)
Excess somatic inpatients (95% CI)	€328 (€202 to €454)	€523 (€399 to €647)	€641 (€354 to €928)	€-211 (€-543 to €122)
Excess somatic outpatients (95% CI)	€193 (€85 to €302)	€131 (€55 to €207)	€304 (€138 to €469)	€-20 (€-231 to €190)
Excess psychiatric inpatients (95% CI)	€27 (€-1 to €56)	€929 (€668 to €1,190)	€2,273 (€1,298 to €3,247)	€1,316 (€300 to €2,333)
Excess psychiatric outpatients (95% CI)	€63 (€41 to €85)	€531 (€467 to €596)	€1,138 (€973 to €1,303)	€543 (€362 to €724)
**Income gap (95% CI)**	€-6,709 (€-7,326 to €-6,093)	€-9,468 (€-9,968 to €-8,969)	€-14,273 (€-15,075 to €-13,471)	€1,905 (€798 to €3,011)
Wage income difference (95% CI)	€-10,523 (€-11,275 to €-9,771)	€-16,584 (€-17,255 to €-15,914)	€-26,305 (€-27,406 to €-25,204)	€802 (€-675 to €2,279)
Excess transfer payments (95% CI)	€3,814 (€3,525 to €4,102)	€7,116 (€6,801 to €7,431)	€12,032 (€11,457 to €12,606)	€1,102 (€385 to €1,820)
Social isolation				
**Excess health care costs (95% CI)**	€668 (€307 to €1,029)	€2,814 (€2,461 to €3,167)	€5,214 (€2,924 to €7,504)	€1,732 (€-623 to €4,088)
Excess GPs and specialists (95% CI)	€23 (€8 to €37)	€137 (€128 to €146)	€171 (€129 to €214)	€12 (€-34 to €58)
Excess subsidised prescriptions (95% CI)	€135 (€95 to €175)	€226 (€191 to €261)	€463 (€358 to €568)	€102 (€-16 to €220)
Excess somatic inpatients (95% CI)	€359 (€95 to €623)	€515 (€392 to €638)	€758 (€299 to €1,218)	€-116 (€-647 to €415)
Excess somatic outpatients (95% CI)	€97 (€-76 to €271)	€165 (€90 to €240)	€136 (€-106 to €378)	€-127 (€-432 to €178)
Excess psychiatric inpatients (95% CI)	€31 (€-9 to €71)	€1,139 (€862 to €1,416)	€2,527 (€423 to €4,631)	€1,357 (€-771 to €3,485)
Excess psychiatric outpatients (95% CI)	€22 (€9 to €35)	€632 (€571 to €693)	€1,158 (€831 to €1,485)	€504 (€171 to €837)
**Income gap (95% CI)**	€-5,387 (€-6,053 to €-4,721)	€-9,703 (€-10,157 to €-9,249)	€-16,461 (€-17,836 to €-15,086)	€-1,370 (€-2,947 to €207)
Wage income difference (95% CI)	€-12,528 (€-13,380 to €-11,675)	€-17,017 (€-17,624 to €-16,410)	€-33,718 (€-35,476 to €-31,960)	€-4,173 (€-6,224 to €-2,123)
Excess transfer payments (95% CI)	€7,141 (€6,739 to €7,543)	€7,313 (€7,028 to €7,599)	€17,257 (€16,405 to €18,110)	€2,803 (€1,796 to €3,810)
Low social support				
**Excess health care costs (95% CI)**	€307 (€190 to €424)	€2,913 (€2,504 to €3,322)	€3,471 (€2,599 to €4,343)	€251 (€-724 to €1,226)
Excess GPs and specialists (95% CI)	€28 (€23 to €34)	€136 (€126 to €146)	€164 (€144 to €183)	€-0 (€-23 to €22)
Excess subsidised prescriptions (95% CI)	€48 (€33 to €62)	€244 (€201 to €287)	€273 (€232 to €314)	€-18 (€-80 to €43)
Excess somatic inpatients (95% CI)	€151 (€69 to €232)	€503 (€385 to €621)	€684 (€382 to €987)	€31 (€-301 to €363)
Excess somatic outpatients (95% CI)	€39 (€-23 to €100)	€166 (€79 to €254)	€166 (€52 to €281)	€-39 (€-191 to €113)
Excess psychiatric inpatients (95% CI)	€8 (€-9 to €25)	€1,237 (€909 to €1,566)	€1,343 (€604 to €2,081)	€97 (€-716 to €910)
Excess psychiatric outpatients (95% CI)	€34 (€22 to €46)	€627 (€553 to €701)	€841 (€716 to €966)	€180 (€32 to €328)
**Income gap (95% CI)**	€-3,558 (€-4,004 to €-3,113)	€-9,597 (€-10,108 to €-9,087)	€-13,750 (€-14,556 to €-12,943)	€-594 (€-1,627 to €440)
Wage income difference (95% CI)	€-5,625 (€-6,143 to €-5,106)	€-16,993 (€-17,677 to €-16,308)	€-24,814 (€-25,936 to €-23,692)	€-2,197 (€-3,599 to €-794)
Excess transfer payments (95% CI)	€2,066 (€1,897 to €2,236)	€7,395 (€7,074 to €7,716)	€11,064 (€10,506 to €11,623)	€1,603 (€931 to €2,275)

S5 Table for: *Social disconnectedness, economic outcomes, and the role of pre-existing mental health conditions: a population-based cohort study*

	Social disconnectedness	Mental health condition	Both	Deviation from additivity
Composite measure				
**Excess health care costs (95% CI)**	€386 (€276 to €497)	€2,474 (€2,075 to €2,874)	€3,984 (€3,248 to €4,720)	€1,123 (€271 to €1,976)
Excess GPs and specialists (95% CI)	€33 (€28 to €38)	€130 (€119 to €141)	€168 (€152 to €183)	€4 (€-15 to €24)
Excess subsidised prescriptions (95% CI)	€63 (€51 to €76)	€201 (€173 to €228)	€338 (€266 to €410)	€74 (€-4 to €152)
Excess somatic inpatients (95% CI)	€176 (€100 to €252)	€533 (€398 to €668)	€603 (€392 to €814)	€-106 (€-363 to €151)
Excess somatic outpatients (95% CI)	€67 (€8 to €127)	€143 (€57 to €230)	€216 (€98 to €334)	€5 (€-149 to €160)
Excess psychiatric inpatients (95% CI)	€9 (€-7 to €24)	€969 (€656 to €1,281)	€1,700 (€1,073 to €2,327)	€722 (€11 to €1,433)
Excess psychiatric outpatients (95% CI)	€37 (€27 to €48)	€499 (€424 to €574)	€959 (€844 to €1,074)	€423 (€282 to €564)
**Income gap (95% CI)**	€-4,336 (€-4,729 to €-3,943)	€-8,970 (€-9,539 to €-8,400)	€-13,918 (€-14,567 to €-13,268)	€-612 (€-1,539 to €315)
Wage income difference (95% CI)	€-7,131 (€-7,591 to €-6,671)	€-15,403 (€-16,152 to €-14,653)	€-25,638 (€-26,534 to €-24,743)	€-3,105 (€-4,337 to €-1,873)
Excess transfer payments (95% CI)	€2,795 (€2,641 to €2,949)	€6,433 (€6,087 to €6,779)	€11,721 (€11,269 to €12,172)	€2,493 (€1,899 to €3,086)

CI: Confidence interval; GPs: General practitioners. Missing data was imputed using multiple imputation by chained equations, and the results are weighted based on register data to represent the population of the included regions in 2013 and 2017. The estimates represent values in 2018 and are adjusted for sex, age (included as a natural cubic spline with five knots), year of survey participation, and country of birth.
