# Supplementary material for: Social disconnectedness, economic outcomes, and the role of pre-existing mental health conditions: A population-based cohort study
Source: PLOS Ment Health. 2025 May 28;2(5):e0000218. doi: 10.1371/journal.pmen.0000218 (PMC12798343; doi:10.1371/journal.pmen.0000218)
Supplement: Attachment — Submitted filename: Response to Reviewers.docx [file pmen.0000218.s012.docx]

**Journal Requirements:**

1. Please provide separate figure files in .tif or .eps format. For more information about figure files please see our guidelines:

<https://journals.plos.org/mentalhealth/s/figures>

<https://journals.plos.org/mentalhealth/s/figures#loc-file-requirements>

We have now provided the figures in .eps format.

2. Please provide an Author Summary. This should appear in your manuscript between the Abstract (if applicable) and the Introduction, and should be 150–200 words long. The aim should be to make your findings accessible to a wide audience that includes both scientists and non-scientists. Sample summaries can be found on our website under Submission Guidelines: <https://journals.plos.org/globalpublichealth/s/submission-guidelines#loc-parts-of-a-submission>.

We have now provided an Author Summary of 198 words in the manuscript, positioned between the Abstract and the Introduction, as requested. We have also provided this summary below and welcome any suggestions for improving the wording.

**Author Summary:**

Individuals who are socially disconnected have a higher risk of adverse health outcomes and mortality, but economic outcomes have not been comprehensively studied. To address this gap, we used data on social disconnection (loneliness, social isolation, and low social support) from a population-based survey conducted in Denmark in 2013 and 2017 among individuals aged 16 years and above. Subsequently, we linked with population-wide administrative data in the following calendar year on economic outcomes (healthcare costs, wage income, and transfer payments) which was available for 158,970 survey participants. We compared the economic outcomes of individuals who were socially disconnected to those of individuals who were not socially disconnected but similar in terms of sex, age, survey year, and country of birth. We found that individuals who were socially disconnected had on average annual excess healthcare costs of €561 to €1,674, lower wage income of €7,207 to €16,295, and excess transfer payments of €2,845 to €8,977. These economic differences varied by sex and age and were greater among individuals with pre-existing mental disorders. This study quantifies the economic disparities faced by socially disconnected individuals which may be of importance for future efforts to improve healthcare prioritization and prevent workforce withdrawal.

3. Please review your reference list to ensure that it is complete and correct. If you have cited papers that have been retracted, please include the rationale for doing so in the manuscript text, or remove these references and replace them with relevant current references. Any changes to the reference list should be mentioned in the rebuttal letter that accompanies your revised manuscript. If you need to cite a retracted article, indicate the article’s retracted status in the References list and also include a citation and full reference for the retraction notice.

We have reviewed the reference list to exclude any retracted or unpublished work and have included information on any published corrections to the cited papers. Please let us know if the Journal identifies any additional corrections or retractions.

**Additional Editor Comments:**

There do not appear to be any significant problems that need to be corrected; there is one point where the author(s) could review the description for appropriateness. The description on lines 301-302 of the Results section seems vague and difficult for the reader to understand. According to the results in Figure 4 and Table S5, the confidence intervals of the deviation from additivity for social isolation and low social support include zero and negative values. Thus, the healthcare costs for the two indicators could be equal to or lower than expected based on the additive interaction. It would be better to state these things clearly.

We agree that clear communication of the results is essential and have modified the text to address the results more nuanced and underscore the uncertainty:

“In the interaction analysis shown in Fig 4, we found that the~~all~~ differences in healthcare costs~~economic outcomes~~ were above that expected based on additive interaction for~~of~~ each indicator of social disconnectedness and a pre-existing hospital-diagnosed mental disorder with deviations ranging from €251 to €1,732. With regard to wage income and transfer payments, we found small deviations above that expected based on additivity, except for wage income according to loneliness, where we found less than expected based on additivity. However, these deviations from additivity were estimated with considerable uncertainty, and the differences in healthcare costs according to social isolation and low social support and in wage income according to loneliness could also be equal to or lower than expected based on additive interaction~~the results were also consistent with lower healthcare costs than expected based on additive interaction for social isolation and low social support~~.“ (p. 14–15)

In addition, the supplementary tables in the footnotes to Figures 1 through 4 are incorrectly numbered. For example, the estimates in Figure 1 are reported in Table S2, not Table S3. Please correct these errors.

We apologize for this oversight which has now been corrected. We have further revised all supplementary material to avoid any remaining errors or ambiguities in layout or text.

**Reviewer Comments:**

Reviewer #1: Thank you for the opportunity to review this manuscript. The authors' study examines the differences in economic outcomes associated with social disconnectedness and its interaction with pre-existing mental disorders. The manuscript is objectively and clearly written, with a well-organized structure. The analysis is detailed and rigorous. Notably, the authors provide a comprehensive analysis of the missing data mechanism, an aspect rarely addressed in published articles. I have only one question for clarification: How is the internal consistency of the measures for social contacts and social support? Which test did you use to evaluate it?

We appreciate your positive feedback on the manuscript. We used data from an existing survey, the Danish National Health Survey, which is widely used for research purposes (1). Thus, for this study, we were restricted to the measures for social disconnection which were applied in the survey in 2013 and 2017.

As mentioned in the manuscript, the measure for loneliness, Three-Item Loneliness Scale, has demonstrated good internal consistency, specifically a Cronbach's α of 0.74 in a subsample from the survey in 2013 and 0.81 in a subsample from the survey in 2017 (2).

With regard to social contacts, no consensus exists on the operationalisation of social isolation (3). However, we constructed an index for social isolation with inspiration from the Berkman-Syme Social Network Index (4). An index is a multidimensional instrument where each item represents a different dimension in contrast to a scale which is unidimensional where each item represents the same underlying construct. Since each item represents a different dimension of social isolation, we would not expect a high internal consistency among the items, nor would we assess the index’s reliability based on this criterion.

With regard to social support, we applied a single-item that was inspired by the MOS Social Support Instrument (5), although this item has not been validated. As this was the only item on social support available in the survey, we were unable to test its internal consistency.

We have highlighted in the ‘Strengths and Limitations’ section that these measures have not previously been validated, and we welcome any suggestions to improve the transparency.

“Second, the measure of social support and social isolation have not been validated, and the measure of social isolation did not include associational activities and voluntary work, whereas~~and~~ the measure of social support focused solely on perceived emotional support (31).” (p. 17)

*References in the response above:*

1.	Christensen AI, Lau CJ, Kristensen PL, Johnsen SB, Wingstrand A, Friis K, et al. The Danish National Health Survey: study design, response rate and respondent characteristics in 2010, 2013 and 2017. Scand J Public Health. 2020;50(2):180–8.

2.	Laustsen LM, Christiansen J, Maindal HT, Plana-Ripoll O, Lasgaard M. The longitudinal relation between loneliness and perceived stress: A structural equation modelling analysis of 10,159 individuals. Scand J Public Health. 2023 Feb 16;14034948231151716.

3.	Prohaska T, Burholt V, Burns A, Golden J, Hawkley L, Lawlor B, et al. Consensus statement: loneliness in older adults, the 21st century social determinant of health? BMJ Open. 2020 Aug 1;10(8):e034967.

4.	Berkman LF, Syme SL. Social networks, host resistance, and mortality: a nine-year follow-up study of Alameda County residents. Am J Epidemiol. 1979 Feb;109(2):186–204.

5.	Sherbourne CD, Stewart AL. The MOS social support survey. Soc Sci Med. 1991;32(6):705–14.
